# Ocular Surface Changes Associated with Face Masks in Healthcare Personnel during COVID-19 Pandemic

**DOI:** 10.3390/life12101491

**Published:** 2022-09-26

**Authors:** Filippo Tatti, Lorenzo Mangoni, Simone Pirodda, Giuseppe Demarinis, Claudio Iovino, Emanuele Siotto Pintor, Germano Orrù, Luigi Isaia Lecca, Marcello Campagna, Gloria Denotti, Enrico Peiretti

**Affiliations:** 1 Department of Surgical Sciences, Eye Clinic, University of Cagliari, 09124 Cagliari, Italy; 2 Multidisciplinary Department of Medical, Surgical and Dental Sciences, Eye Clinic, University of Campania Luigi Vanvitelli, 80131 Naples, Italy; 3 Molecular Biology Service Lab, Department of Surgical Science, University of Cagliari, 09124 Cagliari, Italy; 4 Division of Occupational Medicine, Department of Medical Sciences and Public Health, University of Cagliari, 09042 Monserrato, Italy; 5 Department of Surgical Science, Institute of Dentistry, University of Cagliari, 09124 Cagliari, Italy

**Keywords:** COVID-19, face mask, dry eye, mask-associated dry eye

## Abstract

The aim of this study was to investigate ocular surface changes associated with face mask (FMs) use of healthcare personnel during the COVID-19 pandemic. We prospectively evaluated 200 eyes of 100 individuals during working hours and 40 eyes of 20 individuals during their rest days as a control group. Dry eye symptoms were assessed with the Ocular Surface Disease Index (OSDI) and McMonnies questionnaire. The clinical investigation included the best corrected visual acuity (BCVA), corneal fluorescein staining (FS), break-up time (BUT), and Schirmer test I before and after a 7-h work shift with a continuative use of surgical or N95 masks. The control group was evaluated similarly twice a day, at 8:00 a.m. and at 3:00 p.m.. In the study group, BCVA, FS, BUT, and Schirmer test were investigated and there was a significant negative variation at the end of the shift. On the contrary, the control group did not show significant variations of any clinical feature. Furthermore, no significant changes in clinical parameters were observed during the use of surgical or N95 masks. In conclusion, FMs continuative use resulted in daily ocular surface modifications specifically in healthcare personnel.

## 1. Introduction

Severe acute respiratory syndrome coronavirus 2 (SARS-CoV-2) emerged at the end of 2019, causing severe pneumonia in a number of patients in Wuhan, China. Subsequently, it rapidly spread worldwide, leading the World Health Organization (WHO) to declare the related disease, named coronavirus disease 2019 (COVID-19), a worldwide pandemic by the end of January 2020 [1]. In parallel with the vaccination campaign, proven to be effective at preventing not only severe stages of the disease, but also patients’ hospitalization and death, the global spread of SARS-CoV-2 led to the adoption of urgent measures to contain its transmission [2,3,4]. However, while the imperative use of face masks (FMs) played a pivotal role in avoiding the forward projection of expelled air and preventing airborne virus transmission, several studies reported their significant contribution to the worsening of dry eye disease (DED) symptoms [5,6,7,8] and, consequently, to the quality of life (QoL) [9,10]. Indeed, an increased prevalence of ocular surface modifications, ocular discomfort, and ocular surface homeostasis impairment due to prolonged use of FMs during the COVID-19 pandemic was highlighted in the most recent literature [11,12,13,14]. Indeed, the air expelled when wearing FMs and directed upwards through the top opening of the mask [15,16,17] would wrap around the corneal surface, creating conditions that may accelerate corneal tear film evaporation and lead to a new clinical entity called mask-associated dry eye (MADE) [11,13]. A recent study identified other co-risk factors implicated in DED pathogenesis in the COVID era including digital devices misuse, unbalanced diet, insufficient hydration, sleep deprivation, and the psychological repercussions of pandemic restrictions [5].

Under this light, considering the WHO recommendations on wearing FMs (N95, FFP2, or FFP3) in healthcare settings [18] and the work shifts of health workers (about 7 h/day in Italy), these individuals may potentially be at high risk for the occurrence or worsening of DED. Moreover, even though mask-associated dry eye has already been demonstrated, information on daily ocular surface modifications still remains unknown. Therefore, the aim of this study was to investigate FMs-related ocular surface changes in healthcare personnel, using questionnaires and clinical examinations.

## 2. Materials and Methods

### 2.1. Subjects

This was a prospective study conducted at the University Hospital of Cagliari, Italy, during the COVID-19 pandemic. A total of 100 health workers who wore FMs continuously during their work shifts were enrolled from April to June 2021, for a total of 200 eyes examined. The cohort consisted of 56 physicians and 44 nurses from the ophthalmologic, dermatologic, and occupational health units and the microbiology laboratory. All subjects were evaluated before work, between 07:00 and 08:00 a.m., and at the end of the shift, between 2:00 and 3:00 p.m. Each subject confirmed the continuative use of surgical or FFP2/N95 FMs with ear loops (both made by BYD Precision Manufacture Co. Ltd., Shenzhen, China) and allocated into 2 groups based on the FM type used. A control group of forty eyes of 20 health workers was evaluated twice a day, at 8:00 a.m. and 3:00 p.m., during their rest days.

The exclusion criteria included previous ocular surgery, a history of systemic or intraocular disease, use of topical therapies during work that could modify the ocular surface, and smoking habit. The study adhered to the tenets of the Declaration of Helsinki and the protocol used was approved by the local Institutional Review Board (PG/2021/5479). Informed consent was obtained before enrolment from all participants. 

### 2.2. Questionnaire

All subjects were evaluated for DED at baseline with the Ocular Surface Disease Index (OSDI) and the McMonnies questionnaire. The OSDI is a 12-item scale created to assess subjective dry eye symptoms and the effects of the disease on vision-related activities of daily living within the previous week. The total OSDI score ranges from 0 to 100 points and positively correlates with DED severity, with a cut-off value of 13 for a diagnosis of DED [19]. The McMonnies questionnaire is a 12-item instrument for the screening of dry eye disease. Each question is individually scored and the McMonnies index is calculated by individually summing them up (perfect score = 45). The severity of dry eye symptoms correlates with this index, with 14.5 points as the cut-off value for a diagnosis of DED [19]. All participants were also asked about other co-risk factors, such as exposure to air-conditioning and the use of visual display terminals (VDTs) [9]. 

### 2.3. Clinical Examination

The clinical tests performed included in sequence best-corrected visual acuity (BCVA), anterior segment slit-lamp examination, corneal fluorescein staining (FS), tear film break-up time (BUT), and Schirmer test I. BCVA was measured by using a 3-m Snellen chart and then converted to LogMAR. FS was evaluated according to the Oxford Grading Scale (OGS) on the basis of the fluorescein dye staining pattern on an ocular surface [20]. BUT is evaluated as the timespan, after fluorescein dye application, between a complete blink and the appearance of the first dry spot on the corneal surface. Schirmer test I is performed by folding a Schirmer paper strip at the notch and hooking the folded end over the temporal one-third of the lower lid margin. The score is then measured as the length of wetting from the notch after 5 min with the eyes gently closed. In accordance with previous studies, we considered as pathological a cut-off of 10 s and 10 mm/5 min for BUT and Schirmer test I, respectively [21]. 

### 2.4. Evaluation Method

All subjects were evaluated by 2 independent observers (F.T. and S.P.), who collected all clinical data as well. The series of examinations lasted approximately 15 to 20 min for each patient. The concordant data were assumed as final data; conversely, a mean value was calculated from the discordant data. If one of the observers was not present at the time of the assessment, the visit was recorded on video and subsequently evaluated by the second observer. 

### 2.5. Statistical Analysis

Data were recorded on Microsoft Office Excel (Microsoft Corporation, Redmond, WA, USA) and the statistical analysis was performed using Excel and SPSS Statistics v28.0 (IBM Corp., Armonk, NY, USA). The Kolmogorov–Smirnov test was used to evaluate the normal distribution for each variable. A paired t-test or Wilcoxon rank sum test was used to compare continuous variables before and after the shift. A mixed ANOVA was used to compare continuous variables before and after the shift, with the type of mask used as the between-subjects factor. Multiple regression analysis was performed to investigate the relationship between demographic data and parameter changes. Any p-values less than 0.05 were considered statistically significant.

## 3. Results

A total of 200 eyes of 100 health workers (44 men and 56 women) who wore FMs at work were included. Their mean age was 44.56 years (±14.87). The study participants were evaluated by the OSDI and McMonnies questionnaire to make an accurate assessment of DED. An amount of 33 subjects exceeded the cut-off value of 13 for the OSDI screening, whereas 19 subjects showed positive results (score > 14.5) on the McMonnies questionnaire. The control group of 40 eyes of 20 subjects (8 males and 12 females) showed a mean age of 40.9 (±13.93). All demographic data of the study participants are summarized in Table 1.

### 3.1. Clinical Parameters

**Figure 1:**
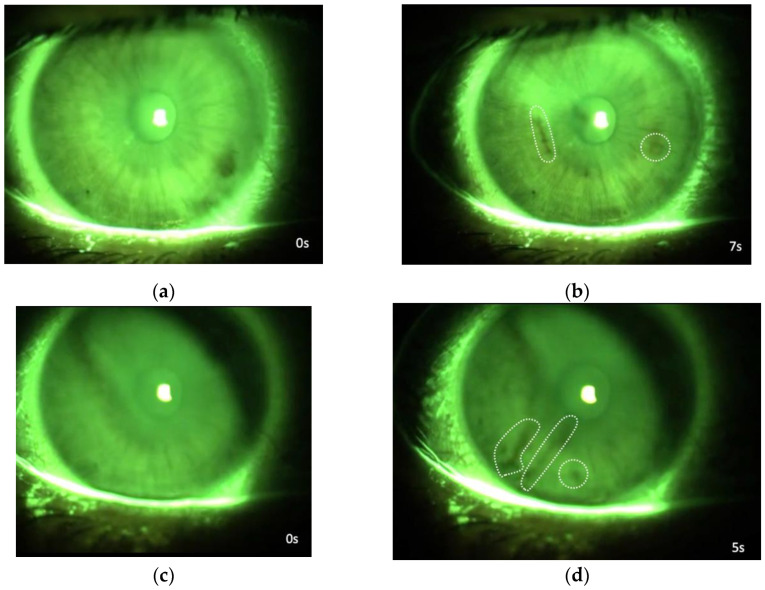
Variations in break-up time (BUT) of a 50-year-old female health worker. Representative slit-lamp frames are from a BUT video of case 44. The first examination (08:05) shows (**a**) stable tear film after blinking at the start of the measurement area and (**b**) the development of dark areas at 7 s. The second examination (14:05) shows (**c**) stable tear film at the start of the measurement area and (**d**) the development of bigger dark areas at 5 s (**d**).

The clinical data collected before and at the end of the work shift are reported in Table 2. Overall, BCVA, BUT (Figure 1), Schirmer test, and FS showed significantly worse values at the end of the shift (all *p* < 0.001). 

There were not any significant differences between the pre-shift clinical parameters of the control group and the pre-shift clinical parameters of the study group (all *p* > 0.05) (Table 3).

As shown in Table 4, the control group showed no significant variation (*p* > 0.05) of any clinical parameter (BCVA, BUT, Schirmer test, and FS). 

### 3.2. DED Diagnosis

The BUT test showed a pathological score of 104 and 153 for eyes before and after work, respectively. With regards to the Schirmer test, it demonstrated an increase in the number of eyes affected from 50 to 76, before and after the work shift, respectively. 

Stratifying the cohort on the basis of BUT and Schirmer test severity and assessing the data before and after the 7-h shift, significant differences were observed (*p* < 0.005) with substantially worse test results at the end of the 7-h shift.

In accordance with the Dry Eye Workshop II guidelines, the presence of dry eye symptoms (OSDI score ≥ 13) with at least one homeostasis test resulting positive, such as BUT < 10 s, allows diagnosis of DED [21]. 

Considering only the shorter BUT of both the eyes of the 33 subjects with a positive result on OSDI screening (score ≥ 13), a clinical diagnosis of DED could be made for 21 and 32 individuals before and after the work shift, respectively (Table 5).

### 3.3. Type of Mask

There were no significant differences between the groups in terms of age, gender, OSDI, McMonnies, time of VDT use, and use of glasses (Table 6). 

**Figure 2:**
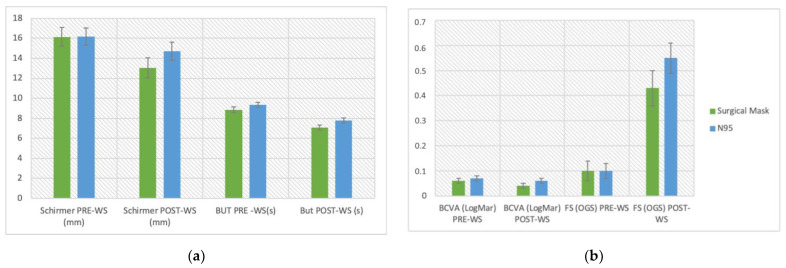
Variations in clinical parameters after continuative use of surgical mask or respirators during work. The bar graph (**a**,**b**) illustrates values of clinical parameters (Schirmer, BUT, BCVA and OGS); bars represent mean values and standard errors (SE) in surgical mask group (green) and N95 group (blue) as indicated in Table 7.

In both groups, there was a significant difference between clinical parameters collected before and after the shift (*p* < 0.05). We also performed a comparative analysis between the groups for BCVA, Schirmer, BUT, and OGS score variance after working with FMs (Figure 2). 

**Table 7:** Variations in clinical parameters after continuative use of surgical mask or N95 during work.

Variations in Clinical Parameters																
	Schirmer (mm)				BUT (s)				BCVA (LogMar)				FS (OGS)			
FACE MASK TYPE	PRE-WS	SE	POST-WS	SE	PRE-WS	SE	POST-WS	SE	PRE-WS	SE	POST-WS	SE	PRE-WS	SE	POST-WS	SE
Surgical Mask	16.14	0.94	13.05	1.02	8.86	0.28	7.06	0.25	0.06	0.01	0.07	0.01	0.1	0.04	0.43	0.07
N95	16.18	0.85	14.70	0.92	9.34	0.26	7.78	0.24	0.04	0.01	0.06	0.01	0.1	0.03	0.55	0.06

BCVA, best corrected visual acuity; BUT, break-up time; FS, Fluorescein staining; OGS, Oxford Grading Scale; WS, work shift.

As shown in Table 8, no significant differences between surgical masks and N95s were observed (*p* > 0.05).

### 3.4. Correlations

Multiple regression analysis between demographic and clinical variations was performed, with weakly significant results (Table 9). The analysis of variance showed that BCVA variation was negatively correlated with age (R = −0.23; *p* = 0.0047) and OSDI score (R = −0.20; *p* = 0.0303), and positively correlated with male sex (R = 0.15; *p* = 0.0187). BUT variation was weakly correlated with time of VDT use (R = −0.08; *p* = 0.0469). All other correlations were not significant.

## 4. Discussion

Although the continuative use of FMs is essential to control the COVID-19 pandemic’s spreading, it may influence ocular surface health owing to air leaking around the mask’s edge during inhalation and exhalation [8,11,13,14,22].

In our study, we investigated ocular surface changes through standard tests, including BCVA, BUT, Schirmer test, and FS [9,23]. In particular, we analysed the variations in these parameters in healthcare workers after a 7-h shift with continuous use of FMs.

Giannaccare et al. suggested that the role of FMs in ocular discomfort symptoms is due to the rapid tear evaporation caused by air dispersion around the mask [11]. Considering that DED is clinically divided into two subtypes based on tear production (deficiency of aqueous or hypo-secretive component) or evaporation (hyper-evaporative) [9], MADE may be included in the second category. 

As previously reported, high airflow may lead to water evaporation from the precorneal tear film with resulting dry spots formation [24,25,26]. Indeed, a significant decrease in lower tear meniscus dimensions was shown in subjects with a baseline short BUT (<5 s) after exposure to an airflow of 1.5  m/s [26]. On the other hand, the airflow effect in healthy eyes is controversial. Some authors demonstrated an increase of lower tear meniscus dimensions as a result of reflex sensory loop and tear secretion compensation induced by airflow exposure changes in the tear film [26]. In contrast, Wyon et al. reported that an exposure of the tear film to high air velocity (1.0 m/s) for 30 min led to a significant decrease in tear stability, as demonstrated by reduced BUT in healthy eyes [24].

Similarly, in our study, there was evidence of significant worsening of the clinical parameters analysed in the entire cohort after wearing FMS continuously during work and exposing the tear film to the related abnormal airflow.

Given the need to wear a FM throughout the whole shift, the ocular surface of healthcare workers’ eyes is subjected to limited airflow for a long time. This condition of chronic ocular surface insult could lead to long-term damage of tear film stability, as demonstrated by Mastropasqua et al. In fact, the continuative use of FMs for more than 6 h/day during a 3-month follow-up, worsened clinical indicators of ocular surface disease and increased cellular and molecular inflammation bio-markers [14]. These signs of ocular surface damage, seen in healthy subjects, were more severe in patients with DED even when they used FMs for less than 6 h/day [14].

The present study shows daily ocular surface changes in healthcare personnel after continuous use of FMs for about 7 h, and we supposed that these daily changes could lead to long-term damage, as shown in the previous study by Mastropasqua et al. [14].

It is important to highlight that the analysis of clinical parameters before and after the work shift could be affected by a physiological modification of tear film during the day, which has been, however, a particularly controversial area of research [27,28,29,30,31]. Oncel et al. reported that tear osmolarity does not have diurnal oscillations in normal subjects [27]. Similarly, no significant differences were found between morning and evening BUT measurements or in tear ferning test patterns at different hours of the day [28,29]. On the contrary, a daily decrease in tear meniscus volume by tear strip meniscometry was reported by Ayaki et al. [30]. A study by Lira et al. [31] showed that tear film quantity, analysed by Schirmer measurements and tear meniscus height, is unrelated to the hour it is assessed, while there is worsening of tear film quality evaluated by BUT and non-invasive BUT. In those studies [28,29,30,31], the authors suggested that the behaviour of tear film is different between patients with diagnosed DED and normal subjects. This concept was in fact confirmed by comparing visual function in the two groups. Walker et al. [32] reported that individuals with dry eyes experience significant diurnal fluctuations in visual function and in signs and symptoms of their condition due to an increased rate of keratitis in the evening, whereas previous research on normal subjects demonstrated an increase in visual function ability from morning to evening [33]. Our results are consistent with this finding, showing a BCVA worsening at the end of the shift, with wearing FMs possibly playing a pivotal role in that phenomenon.

Although all FMs are effective at slowing down the frontward flow and the horizontal distance travelled by aerosol particles [15,16], a surgical mask not fitting the face tightly causes greater air leakage around the sides during coughing [15], whereas an upward leakage jet was described for FFP2 masks when it was not possible to seal them properly [15]. In our study, no demographic differences were found between the groups of surgical masks and N95 wearers. Nevertheless, a worsening of the clinical parameters was found in both the groups at the end of the work shift that we supposed to be related to an upward dispersion of exhaled air. We suggest that the mask should be carefully shaped to the nose to ensure a proper seal and, in turn, to reduce the airflow over the corneal surface.

Although Krolo et al. [12] observed a higher incidence of MADE in women and subjects with a previous history of DED who wore FMs longer than 3 h/day, in the present study, we did not find any significant correlation either between parameter variations and demographic data nor between dry eye assessment (OSDI and McMonnies questionnaire) and ocular surface changes. However, it is interesting to note that among the 33 healthcare workers with dry eye symptoms, 21 showed BUT impairment (<10 s) before and after work while 11 of them showed impairment only after work. Only one individual did not show any homeostasis biomarker alteration.

With regards to the use of goggles or eye protection devices, these were reported to increase periocular humidity and therefore could be a protective measure for dry eye occurrence or worsening [34,35]. However, in the present study, wearing glasses or eye protection equipment was found to not influence the variation of parameters explored.

WHO estimates that between 80,000 and 180,000 healthcare workers died from COVID-19 between January 2020 and May 2021, converging to a medium scenario of 115,500 deaths [36]. The severity of the complications related to COVID-19 underlines the importance of using preventive measures, especially in healthcare settings. Indeed, face masks are an essential tool for healthcare personnel, and their use is mandatory due to the more concerning consequences of SARS-COV-2 infection more than dry eye symptoms [18]. Nevertheless, long-term mask wearing has created a new problem to cope with, albeit of minor importance.

Defining an occupational disease involves two main elements: the exposure–effect relationship between a specific working environment and/or activity and a specific disease effect, and the fact that these diseases occur among certain groups with a frequency higher than the average for the rest of the population [37]. Further study is required to define whether those criteria will be met and affect the magnitude of DED in mask wearers worldwide.

To our knowledge, the present is the first study to focus on ocular surface daily modifications in healthcare personnel wearing FMs. The short-term follow-up better reflects the ocular surface damage induced by only using FMs.

Limitations of the current study include the lack of close monitoring of the subjects during the work shift to ensure proper mask sealing. In addition, study participants were asked about the use of video terminals, but no specific environment information were evaluated: further study should be directed to also investigate humidity levels and room lighting of each workday environment. Furthermore, the two study arms were not homogeneous in terms of number: the control group of subjects not using FMs was smaller than the study group. Another limitation was that we exclusively assessed the FMs’ influence with standard tests of DED, while a tear meniscus analysis with optical coherence tomography, osmolarity testing, and the identification of pro-inflammatory markers may add useful information.

Further population studies involving larger cohorts will be required to better define the actual incidence and severity of DED in mask wearers worldwide. Given the intensive FMs use, nowadays compulsory in many different settings, and the fact that DED often remains unrecognized until symptoms are significantly advanced [7], a DED assessment to evaluate the need for therapy should be performed as part of work health control. On the other hand, healthcare personnel should observe a series of precautions to reduce the impact of FMs on their ocular surfaces, such as ensuring the mask to be properly sealed onto the nose reducing the airflow turbulence towards the eyes. In addition, following simple healthy lifestyle measures, such as drinking an adequate amount of water to counteract the drying process, may be appropriate.

Furthermore, considering the difficulty of predicting the duration of anti-pandemic measures, a longitudinal examination of DED signs and symptoms in healthy subjects and DED patients would be desirable to evaluate the long-term impact of these measures on ocular surfaces.

## 5. Conclusions

FMs are an essential tool for healthcare personnel, and their use is mandatory to avoid concerning consequences of SARS-COV-2 infection [18]. However, wearing them for long-term periods, especially in work environments, has created several minor issues, including modifications to the ocular surface and clinical signs of DED. The worsening of clinical parameters did not correlate with the type of FM worn. Further studies are required to avoid potential biases and identify ocular surface inflammation markers.

## Figures and Tables

**Table 1:** Demographic characteristics of the sample of health workers.

	Study Group = 100		Control Group = 20		
Parameter	Value	SD	Value	SD	*p*
Mean age (years)	44.56	14.87	40.9	13.93	*0.069*
Sex					
M	44 (44%)		8 (40%)		*0.74*
F	56 (56%)		12 (60%)		
Mean OSDI	12.41	12.06	12.57	12.78	*0.82*
Mean McMonnies	10.22	5.25	11.28	5.38	*0.53*
Mean VDT use (h)	0.60	0.95	0.7	0.86	*0.47*
Glasses/eye protection					
Yes	49 (49%)				
No	51 (51%)				

SD, standard deviation; VDT, video display terminal.

**Table 2:** Clinical parameters collected before and at the end of the work shift.

Parameter	Before Work Shift	SD	End of Work Shift	SD	*p*-Value
Mean BCVA (LogMar)	0.05	0.09	0.07	0.10	*<0.001*
Mean Schirmer test value (mm)	16.16	8.90	14.04	9.70	*<0.001*
Mean BUT (seconds)	9.15	2.67	7.49	2.53	*<0.001*
Mean FS (OGS)	0.10	0.35	0.51	0.66	*<0.001*

BCVA, best corrected visual acuity; BUT, break-up time; FS, Fluorescein staining; OGS, Oxford Grading Scale; SD, standard deviation.

**Table 3:** Clinical parameters collected at 8:00 a.m. in the control group and in the study group.

Pre-Shift Parameter	Control Group	Study Group	*p*-Value
**Mean BCVA (LogMar)**	0.025	0.05	0.06
**Mean Schirmer Test Value (mm)**	18.68	16.16	0.12
**Mean BUT (seconds)**	10.38	9.15	0.24
**Mean FS (OGS)**	0.15	0.1	0.22

BCVA, best corrected visual acuity; BUT, break-up time; FS, Fluorescein staining; OGS, Oxford Grading Scale; SD, standard deviation.

**Table 4:** Clinical parameters collected in the control group.

Parameter	8:00 a.m. Examination	SD	3:00 p.m. Examination	SD	*p*-Value
**Mean BCVA (LogMar)**	0.025	*0.07*	0.01	*0.03*	0.09
**Mean Schirmer Test Value (mm)**	18.68	*6.94*	18.35	*7.34*	0.69
**Mean BUT (seconds)**	10.38	3.64	10.25	*3.40*	0.59
**Mean FS (OGS)**	0.15	*0.36*	0.2	*0.40*	0.15

BCVA, best corrected visual acuity; BUT, break-up time; FS, Fluorescein staining; OGS, Oxford Grading Scale; SD, standard deviation.

**Table 5:** Study group for the dry eye disease assessment. In brackets, tests results are expressed in mean (±SD).

	N. Eyes (Mean Values ± SD)	
**Schirmer Test**	Pre-WS	Post-WS
<10 mm/5 m	N. Eyes: 50 (4.9 ± 1.56)	N. eyes: 76 (4.32 ± 4.51)
<5 mm/5 m	N. Eyes: 9 (2.44 ± 1.24)	N. Eyes: 39 (1.56 ± 1.33)
**BUT**		
<10 s	N. Eyes: 104 (7.01 ± 1.65)	N. Eyes: 153 (6.17 ± 1.97)
5–10s	N. Eyes: 95 (7.35 ± 1.26)	N. Eyes: 130 (6.46 ± 1.79)
<5 s	N. Eyes: 9 (3.44 ± 0.72)	N. Eyes: 23 (3.11 ± 0.92)
	**Subjects**	
**DED symptoms** **(OSDI score ≥ 13)**	33	
Mild (13–22)	17	
Moderate (23–32)	8	
Severe (33–100)	8	
**DED Diagnosis** **(OSDI score ≥ 13 and BUT < 10 s)**	21	32

N., Number of; SD, standard deviation; WS, work shift; DED, dry eye disease; BUT, break-up time.

**Table 6:** Demographic parameters of surgical mask and N95 groups.

	Surgical Mask Group (n = 40)	N95 Group (n = 60)	*p*-Value
Age (y)	45.2 ± 14.7	44.1 ± 15.1	0.72
Gender (M:F)	20:20	24:36	0.32
OSDI	9.5 ± 9.2	13.8 ± 13.6	0.06
McMonnies	9.2 ± 4.7	10.9 ± 5.5	0.10
VDT use (h)	0.7 ± 1.1	0.6 ± 0.8	0.61
Wearing glasses	17	34	0.16

VDT, video display terminal.

**Table 8:** ANOVA with repeated measures analysis shows a statistically significant difference between variables pre/post continuative use of FMs (*p* < 0.001). Face mask type (surgical or respirator) had no statistically significant effect on clinical parameters after work (*p* > 0.05).

Test of within-Subjects Effect								
	Schirmer (mm)		BUT (s)		BCVA (LogMar)		FS (OGS)	
SOURCE	F (1,198)	*p*-Value	F (1,198)	*p*-Value	F (1,198)	*p*-Value	F (1,198)	*p*-Value
Pre/Post Work Shift	23.54	<0.001	132.60	<0.001	23.60	<0.001	12.16	<0.001
Face Mask Type	0.48	0.49	2.57	0.111	0.69	0.41	2.79	0.96

FMs, face masks; BCVA, best corrected visual acuity; BUT, break-up time; FS, Fluorescein staining; OGS, Oxford Grading Scale.

**Table 9:** Multiple regression analysis between demographic and clinical parameters.

Parameter	Age (y)	Gender	OSDI Score	McMonnies Score	VDT Use Time (h)	Wearing Glasses
**BCVA Variation** **(LogMar)**	−0.23 (*p* = 0.0047)	0.15 (*p* = 0.0187)	−0.20 (*p* = 0.0303)	−0.13 (*p* = 0.1333)	−0.02 (*p* = 0.7077)	−0.07 (*p* = 0.1975)
**Schirmer Test Variation** **(mm)**	0.09 (*p* = 0.2212)	−0.11 (*p* = 0.2265)	0.05 (*p* = 0.1921)	0.04 (*p* = 0.7628)	0.12 (*p* = 0.1543)	−0.019 (*p* = 0.6985)
**BUT Variation (s)**	−0.08 (*p* = 0.0833)	−0.04 (*p* = 0.6917)	0.02 (*p* = 0.5643)	−0.12 (*p* = 0.2213)	−0.08 (*p* = 0.0469)	0.05 (*p* = 0.6157)
**FS Variation (OGS)**	−0.11 (*p* = 0.0859)	0.06 (*p* = 0.4098)	0.11 (*p* = 0.3813)	0.08 (*p* = 0.1472)	−0.05 (*p* = 0.1970)	−0.11 (*p* = 0.1019)

Y, years; s, seconds; mm, millimetres; h, hours; BCVA, best corrected visual acuity; BUT, break-up time; VDT, video display terminal.

## Data Availability

Not applicable.
